# A Preliminary Color Study of Different Basil-Based Semi-Finished Products during Their Storage

**DOI:** 10.3390/molecules27072059

**Published:** 2022-03-23

**Authors:** Federica Turrini, Emanuele Farinini, Riccardo Leardi, Federica Grasso, Valentina Orlandi, Raffaella Boggia

**Affiliations:** Department of Pharmacy, University of Genoa, Viale Cembrano 4, 16148 Genoa, Italy; emanuele.farinini@edu.unige.it (E.F.); leardi@difar.unige.it (R.L.); grasso@difar.unige.it (F.G.); valentina.orlandi@edu.unige.it (V.O.); boggia@difar.unige.it (R.B.)

**Keywords:** basil, basil semi-finished products, color analysis, stability study, chemometrics

## Abstract

Basil-based semi-finished products, which are mainly used as an intermediate to produce the typical pesto sauce, are prepared and exported all over the world. Color is a fundamental organoleptic requirement for the acceptability of these semi-finished products by the manufacturers of the pesto sauce. Some alternative formulations, which adjust the typical industrial recipe by both changing the preservative agent (ascorbic acid, citric acid, or a mixture of both) and introducing a preliminary thermic treatment (blast chilling), were evaluated. In this work, a fast and non-destructive spectrophotometric analysis, to monitor the color variations in these food products during their shelf-life, was proposed. The raw diffuse reflectance spectra (380–900 nm) obtained by a UV–visible spectrophotometer, endowed with an integrating sphere, together with the CIELab parameters (L*, a*, b*) automatically obtained from these, were considered, and elaborated using multivariate statistical analysis (principal component analysis). From this preliminary study, blast chilling, together with the use of ascorbic acid, proved to be the best solution to better preserve the color of these products during their shelf-life.

## 1. Introduction

Nowadays, some food companies prepare and export basil-based semi-processed products all over the world. These products are designed for use in various sectors of the food industry, such as in restaurant and catering chains and/or gastronomy. These semi-finished products are primarily used as intermediates for pesto sauce production.

Pesto is a typical Italian basil-based pasta sauce, which is common on the international market [1]. It is the second most popular condiment for pasta after tomato sauce [2], and it is widely used all over the world for the preparation of many dishes. Many food industries are working to improve its preparation and conservation processes [3]. This traditional sauce originates from Liguria, a coastal region of Northwestern Italy, overlooking the Mediterranean Sea, which stretches from Tuscany to the French border. Other traditional ingredients of pesto are cheese (most often Parmesan and Pecorino cheese), oil (generally extra virgin oil), garlic, and pine nuts. Pesto is a raw sauce, as the ingredients are not cooked, and, thus, they maintain their own organoleptic characteristics, such as color and flavor. In particular, Ligurian pesto has peculiar organoleptic features, resulting both from the distinctive sensorial character of the P.D.O. (Protected Designation of Origin) Genoese basil (*Ocimum basilicum* L.) [4,5] and from a balanced contribution of all the employed ingredients. The European certification of the Genoese basil P.D.O. defines this excellent Italian agri-food as an “inimitable” product, thanks to its environment of origin and to the traditional techniques handed down over the years, which expresses the perfumes and healthiness typical of the Mediterranean diet [6].

After the basil cutting step, industrial processing, which consists of some accurate washing phases that lead to the raw material being extremely cleaned, takes place. After washing, and then drying, the basil is processed to become a semi-finished product. Small quantities of preservative agents (generally ascorbic acid) are usually added to these semi-finished products to prevent oxidation, by lowering the pH and inactivating some enzymes [7].

Basil-based semi-finished products are highly appreciated by pesto sauce producers for their bright green color, since this feature mainly influences the consumer’s acceptability of the final product. Indeed, color is a fundamental property of food products, and plays an important role in determining the quality of the product itself, and in the decisions of the consumer [8]. However, during storage, these attributes can decrease, sometimes reaching unacceptable levels compared to the corresponding fresh ingredients. As far as color is concerned, it can range from bright green, which is very attractive to the consumers, to a dark brownish green, which, on the contrary, is not an indicator of a fresh and appetizing food [9].

Chlorophylls (chlorophyll a and b) are mainly responsible for the green color of basil-based semi-finished products, and they are chemically formed by a tetrapyrrole system that coordinates a magnesium atom [2]. These pigments are particularly sensitive to chemical–physical variations, such as temperature, pH, the presence of enzymes, and metallic traces, which can lead to modifications of the original color, due to the transformation of chlorophylls into their respective brownish pheophytins [10,11].

The objective of the present work was to monitor the color of the basil-based semi-finished product of an agricultural company located in Western Liguria during its shelf-life, namely, 3 months of storage at refrigerated temperature (4 °C). Moreover, some alternative formulations, which adjusted the typical industrial recipe, considering the company indications, by changing the preservative agent or introducing preliminary blast chilling, were proposed to limit the color variation in this product during its shelf-life.

A new rapid analytical approach, based on the spectrophotometric study of color coupled with multivariate statistical analysis, was explored [12,13]. The raw diffuse reflectance spectra recorded by a UV–visible absorption spectrophotometer, endowed with an integrating sphere, were considered. The results obtained were compared with the traditional approach based on the use of the CIE/CIELab colorimetric parameters (L*, a*, b*), which represent the most common color space [14], and which were automatically supplied by the instrument, starting from the raw spectral data recorded by the software, and then elaborated by chemometrics.

The spectral data analysis was performed using principal component analysis (PCA), as an unsupervised pattern recognition technique, to extract useful analytical information, and to perform dimensionality reduction using a limited number of significant PCs as new variables to describe the samples [15,16,17].

**Figure 1:**
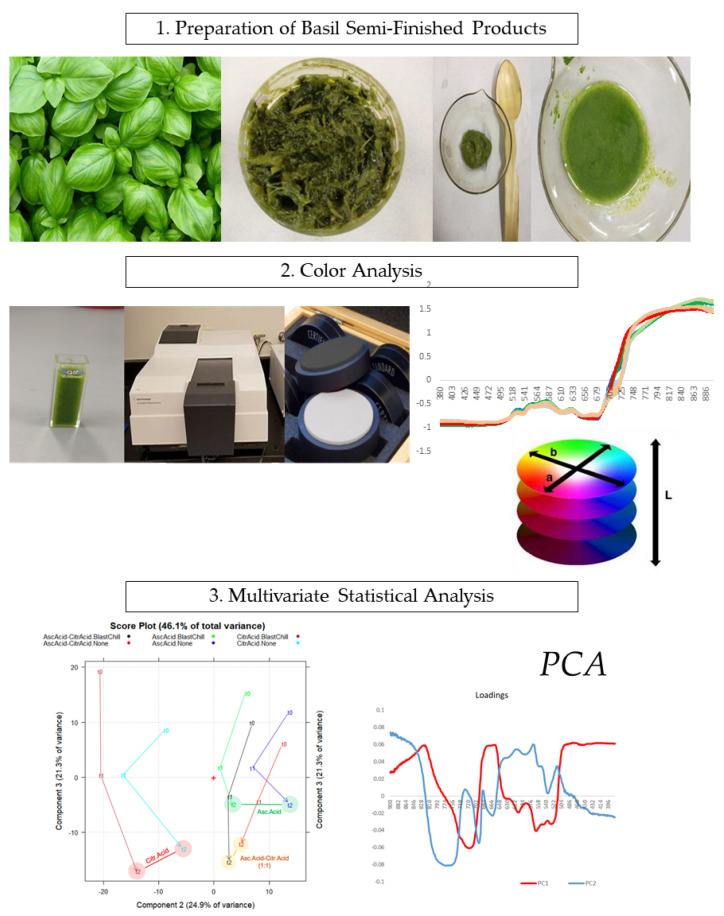
The color study of basil-based semi-finished products.

The global scheme of the present work is reported in Figure 1.

## 2. Results and Discussion

The color of basil, and of vegetables in general, is affected by several parameters, including cultivar, basil geographic origin, geo-pedoclimatic conditions of cultivation, age of the plant, etc. [18].

It is known that in fresh leafy vegetables, the operation of cutting may stimulate enzymatic browning, with important commercial consequences [19]. Basil is subjected to simple operations soon after harvest, such as cleaning, washing, drying and cutting to become a semi-finished product. Cutting is the main factor responsible for the deterioration of these products during storage. Enzymatic browning mostly occurs in fruit and vegetable products during harvesting, transportation, storage, and processing; consequently, it influences the sensory and nutritional values of the derived food products. The chemical methods used to inhibit enzymatic browning include acidification or reduction using antioxidants, chelating agents, or natural extracts. Ascorbic acid is widely used as an anti-browning agent in food samples. The mechanism underlying the anti-browning activity of ascorbic acid appears to rely on its reducing activity [20]. Although ascorbic acid does not directly interact with the polyphenol oxidase enzyme, it inhibits enzymatic browning by reducing oxidized substrates. Recent studies have also suggested that the anti-browning function of ascorbic acid can be attributed to the reduction of enzymatically formed o-quinones to their precursor diphenols, thus preventing the synthesis of the brown pigments responsible for color changes [21].

In this research, to stabilize the color of the basil semi-finished products during their entire storage period (3 months at a refrigeration temperature of 4 °C), several possible alternatives for formulation and storage were evaluated. Primarily, instead of the traditional use of ascorbic acid as an antioxidant agent, it was decided, in accordance with the company’s requirements, to study its total replacement with citric acid, or its partial replacement with a mixture of ascorbic acid and citric acid (1:1). Specifically, ascorbic acid has been replaced with citric acid, since it is one of the best known, safe, and cheap antioxidant food additives (E330) naturally contained in many food products [Regulation (EU) n. 234/2011]. Concurrently, it was assessed whether a preliminary blast chilling treatment (−20 °C), normally not carried out at the company level, could at least partially block the browning by reducing the color alterations of the finished product. Finally, to study ageing, the samples were analyzed every month (up to 3 months, as indicated by the company).

The effect of temperature on the color degradation of green vegetables, as well as the chlorophyll content, as a factor that contributes to color variation, has previously been reported in the literature. Manolopoulou et al. (2016) evaluated the effect of different storage conditions on the color changes of green vegetables, at temperatures of 0 °C, 5 °C, 10 °C, and 20 °C [22]. They showed that vegetables kept at 0 °C better maintain their green color, while increasing the temperature causes aging effects and degrades the chlorophylls. Zeppa et al. (2014) [9] described the changes in color related to temperature increases that occur in a complex food matrix, such as pesto sauce, due to the degradation of the pigments of fresh basil, such as chlorophylls, which are particularly sensitive to degradation reactions.

To study the effect of the above-mentioned variables, the experimental plan was chosen using the experimental design (DoE). The following tables (Table 1 and Table 2) list all the experimental variables considered and the respective coded levels.

The experimental plan, as reported in Table 2, was carried out by considering all the possible combinations, i.e., 2 × 3 × 3 (18 samples); each sample was analyzed using two different cuvettes (18 × 2 = 36) to assess a possible “cuvette effect” in the analytical phase. Each measurement was performed in triplicate (36 × 3 = 118).

The spectra of the 36 analyses obtained are observed in the spectral range 380–900 nm in the visible spectrum area (521 variables), in reflectance.

**Figure 2:**
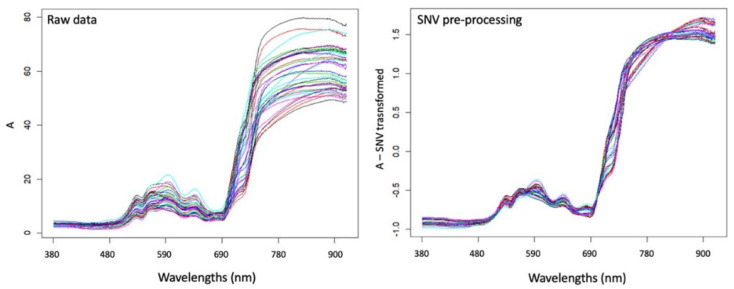
The spectra before and after SNV normalization.

The standard normal variate (SNV) method performs normalization of the spectra, which consists of subtracting each spectrum by its own mean and dividing it by its own standard deviation. After SNV, each spectrum will have a mean of zero and a standard deviation of one (Figure 2). SNV attempts to make all the spectra comparable, in terms of intensities (or absorbance level), and it is important to reduce the scattering effects present in the original spectra [23,24].

To interpret the results and to give a physical meaning, the CIELab coordinates (directly extracted from the instrument) were used to check the presence of trends within the scores.

### 2.1. Color Analysis: Step 1

The first elaboration was performed by considering all the analyses (36 objects; 521 variables, A_36,521_ data matrix) to verify the possible presence of a cuvette effect. PCA, a data display method used in multivariate statistics for the exploratory analysis of a multivariate data set, was applied. The main purpose of PCA is the rationalization of the information contained in the complex data set, the interpretation and processing of useful information, and the elimination of useless noise. Indeed, if you have more than three variables in your data sets, it could be very difficult to visualize a multi-dimensional hyperspace. For these reasons, PCA is used to extract the important information from a multivariate data table, and to express this information as a set of a few new variables, called principal components [25,26]. The main advantages of PCA, compared to a univariate approach, are that it allows high-quality information to be extracted from less-resolved data, and that it provides speed in obtaining real-time information from data, by taking into account the inter-relations between all the variables [27].

**Figure 3:**
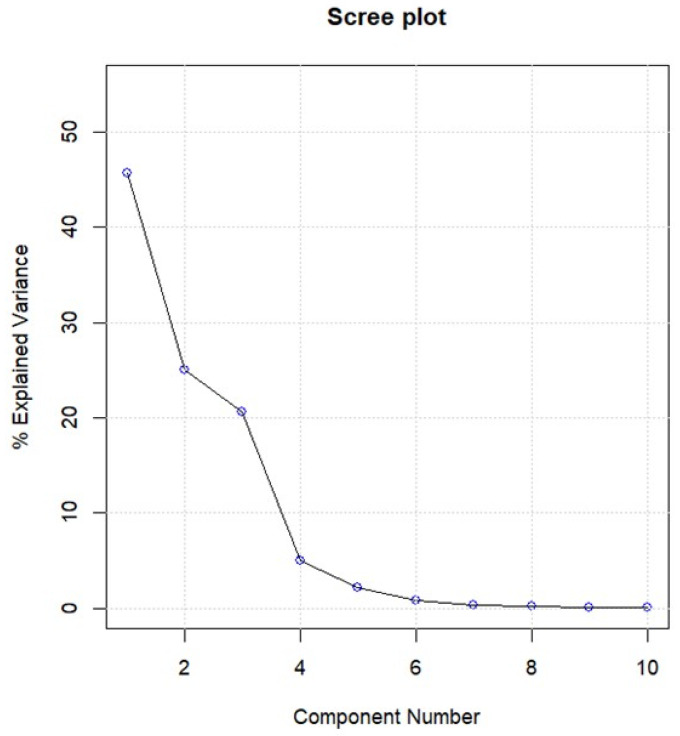
Scree plot of A_36,521_ data matrix.

The PCA performed on the data set in question identifies three significant components that explain 45.6%, 25.0% and 20.6% of the variance (91.2% in total), respectively, as reported in the Scree plot (Figure 3).

**Figure 4:**
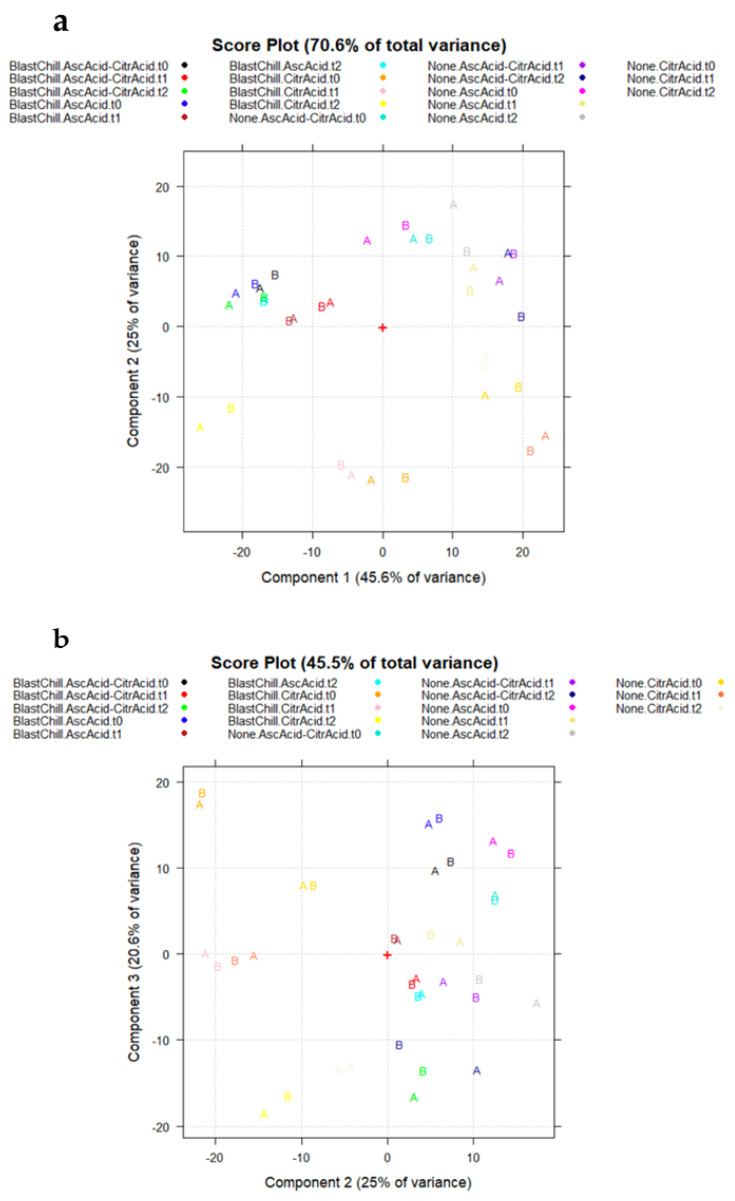
Score plots of A_36,521_ data matrix: (**a**) PC1–PC2; (**b**) PC2–PC3.

Based on the representation of the score plots of A_36,521_ on the planes PC1–PC2 (70.6% total variance explained; Figure 4a) and PC2–PC3 (45.5% total variance explained; Figure 4b), with the objects coded according to the cuvette (A and B, respectively) and colored according to the experimental conditions, it is clear that the “cuvette effect” was totally absent, since the results obtained using the two cuvettes of the same sample are very close to each other in the principal component space. The two clouds (corresponding to A and B, respectively) are completely overlapped; thus, they are not significantly different (Figure 4a,b).

Because of these results, the elaboration was repeated on the average of the two different measurements.

### 2.2. Color Analysis: Step 2

**Figure 5:**
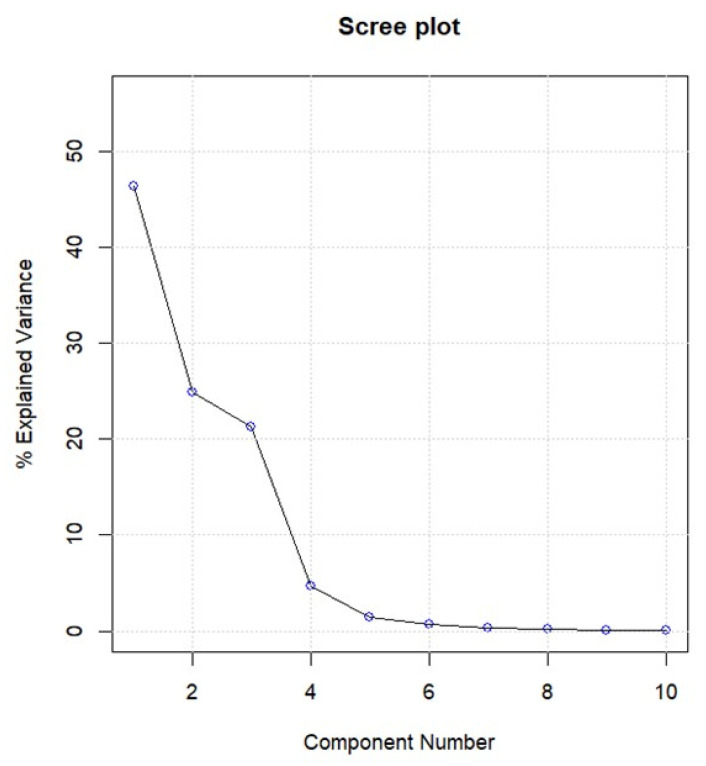
Scree plot of B_18,521_ data matrix.

The PCA performed on the data set (18 objects; 521 variables, B_18,521_ data matrix) in question identified three significant components that explain 46.3%, 24.9% and 21.3% of the variance (92.5% in total), respectively, as reported in Figure 5.

**Figure 6:**
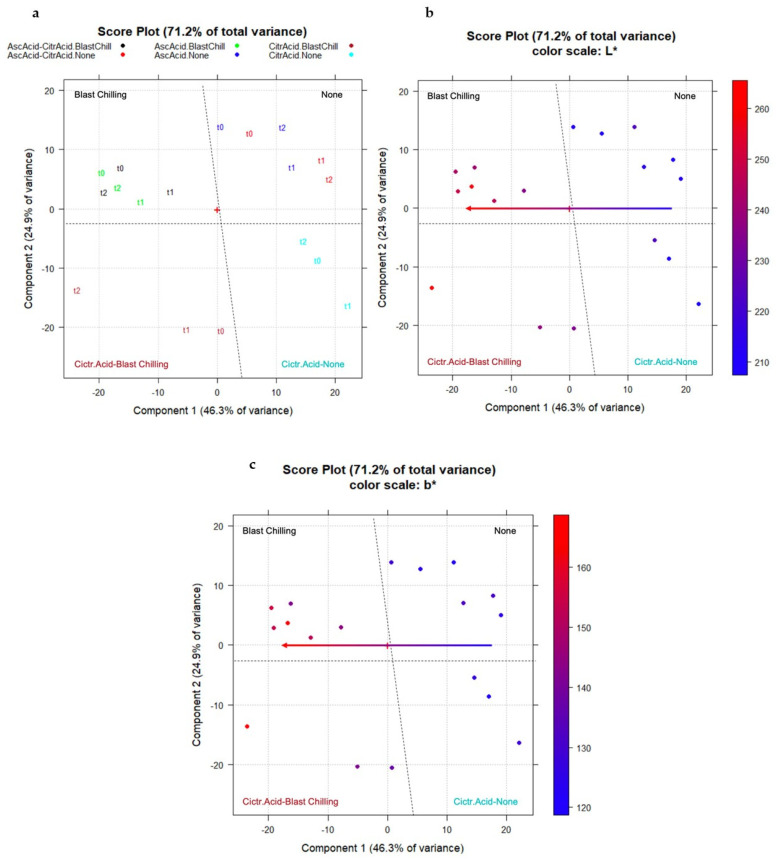
The score plot PC1–PC2 of data matrix B_18,521_. (**a**) The objects were coded according to the time point (t0, t1, and t2) and colored according to the different additive used in the formulation (ascorbic acid, citric acid, or the mixture of ascorbic acid and citric acid); (**b**) the correlation between the L* coordinate (lightness) and PC1 scores is highlighted; (**c**) the correlation between the b* coordinate (bluish–yellowish) and PC1 scores is highlighted.

From the score plot of B_18,521_ in the plane PC1–PC2 (71.2% total variance explained of the whole data set), with the objects coded according to the time point (t0, t1, and t2) and colored according to the different additive in the formulation, it is noticeable that the samples are well characterized by a precise position on the score plot (Figure 6). As highlighted in Figure 6a, PC1 explains the information connected to the chilling treatment, with the treated samples at negative scores (left-hand side of the plot) and the untreated samples at positive scores (right-hand side of the plot). On the other hand, PC2 explains the additive effect, with the citric acid formulated samples well separated at negative scores and the others at positive scores. Then, the CIELab coordinates were considered. The correlation between the L* coordinate (lightness) and the scores on PC1 is clear, where brighter samples are characterized by negative scores (left-hand side of the plot, blast chilled samples) (Figure 6b). The scores on PC1 are also correlated with the b* coordinate (bluish–yellowish), where the yellowish samples are characterized by negative scores (left-hand side of the plot, Figure 6c), leading to the conclusion that L* and b* are very much correlated. PC2, however, is not connected to any of the CIELab coordinates.

**Figure 7:**
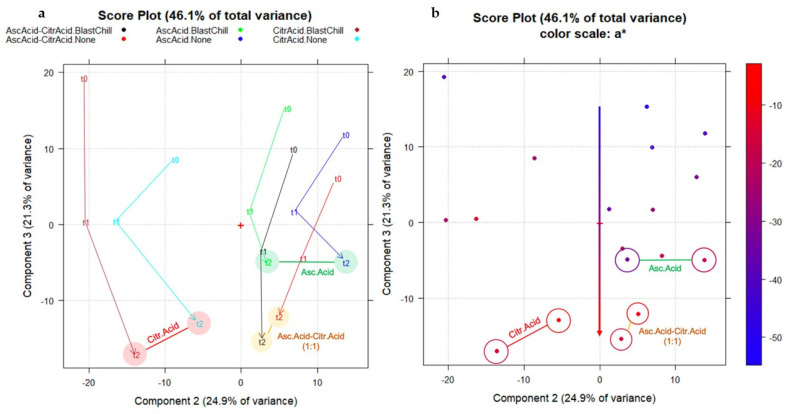
The score plot of data matrix B_18,521_ in the plane PC2–PC3. (**a**) The objects were coded according to the time point (t0, t1, and t2), colored and line-connected according to the additive used in the formulation (ascorbic acid, citric acid or the mixture of ascorbic acid and citric acid); (**b**) the correlation between the a* coordinate (greenish–reddish) and PC3 scores was highlighted.

From the score plot of data matrix B_18,521_ in the plane PC2–PC3 (46.1% total variance explained), with the objects coded according to the time point (t0, t1, and t2), and colored and line-connected according to the additive in the formulation, it is possible to describe the time effect along the third component (PC3). Particularly, the samples at time point t0 are characterized by positive scores (right-hand side of the plot), while the subsequent time points (t1 and t2) are characterized by more negative scores (left-hand side of the plot, Figure 7a). Comparing these results with the a* coordinate (greenish–reddish), the correlation with the scores on PC3 is evident; through the process of ageing, the samples tend to be browner, as the red component prevails over the green component, defined by negative scores on PC3 (Figure 7b). Zeppa et al. (2014) [9] and then Zardetto et al. (2020) [3] performed the color measurement, evaluating the CIELab parameters of fresh green pesto samples during different storage conditions. As reported by the authors, the a* parameter (D65) was the most significant, decreasing during storage, strongly dependent on the temperature conditions. Among the color indices used, the a* (D65) parameter was used to define mathematical functions that allow the color change of the product to be predicted during heat treatments.

Furthermore, thanks to PC2 information (additive effect), a comparison between the different additives through the ageing process is possible; the presence of citric acid in the formulation results in worsening of the color compared to ascorbic acid alone, as the samples with citric acid are all characterized by more negative scores on PC3 (browner color) after 3 months. It is worth mentioning that the samples treated with ascorbic acid, checked at 3 months, appear similar in color to those treated with citric acid, checked after 1 month. This leads to the conclusion that ascorbic acid alone appears to be the preferable additive among those investigated, as it is useful for slowing down the browning process of the semi-finished products.

## 3. Materials and Methods

### 3.1. Preparation of Basil Semi-Finished Products

The basil semi-finished products were prepared starting from fresh leaves of basil P.D.O. grown in Liguria (Albenga, Savona, Italy) and originating from the same collection. Basil was previously washed and sanitized with water and a small amount of hypochlorite, and then rinsed with plenty of water, and partially dried. The semi-finished basil preparations were obtained by grinding in a mill (Grindomix 200 M, Retsch, Haan, Germany) for 20 s at 5000 rpm, with sunflower oil (from certified suppliers whose entire production chain is known), salt, and 0.5% antioxidant agent, namely, L-ascorbic acid, citric acid, or L-ascorbic acid/citric acid 50/50 *w/w*, as reported in Table 3. Immediately after preparation, some semi-finished products (using the three different preservative agents shown in Table 3) were blast chilled at −20 °C for 48 h and subsequently stored at 4 °C, similarly to the other products, before their further analysis. Before the spectrophotometric analysis, a preliminary homogenization step was necessary, as the shredding carried out by the company was uneven and coarse, and this could give rise to scattering problems, invalidating the results of the analysis. Homogenization was carried out using an Ultra-turraxT25 homogenizer for 30 s (5 s at 8000 rpm and 25 s at 24,000 rpm), avoiding temperature rises.

### 3.2. Chemicals

L-ascorbic acid and citric acid, added as preservative agents in basil semi-finished products, were purchased from Sigma-Aldrich (Milan, Italy). High-purity water (18 MΩ) was produced by a Milli-Q system (Millipore, Bedford, MA, USA).

### 3.3. Color Analysis

A double-beam UV–visible spectrophotometer Agilent Cary 100 (Varian Co., Santa Clara, CA, USA) equipped with an integrating sphere (Varian DRA), able to diffuse the light so that it is evenly distributed over its entire internal surface, was employed to evaluate diffuse reflectance. Particularly, UV–visible spectra were recorded in the range between 380 and 900 nm, with a resolution of 0.5 nm, using a white Spectralon^®^ disk as reference. Two quartz cuvettes SUPRASIL^®^300 (Hellma Mullheim, Müllheim, Germany) with rectangular-section cells, a 1 cm path length and a 3.5 mL volume capacity were employed to evaluate the cuvette variability. Samples were acquired randomly, and six replicates of the diffuse reflectance for each sample were recorded and averaged to minimize unwanted spectral variability. The CIELab coordinates L* (lightness), a* (greenish–reddish), and b* (bluish–yellowish) of all samples analyzed were automatically calculated from the raw spectral data by the Cary100 color software using the CIE D65 illuminant. The colorimetric analysis was carried out immediately after the preparation of the semi-finished products (t0) and after 1 and 3 months of refrigerated storage, respectively, which correspond to the monitoring times of interest for the company. For each analysis, a new product package was opened.

### 3.4. Multivariate Statistical Analysis

Principal component analysis (PCA) is the most used tool in exploratory data analysis, to simplify and visualize data by extracting only the important information from the data set [28,29]. Given a data set in which each sample is described by *n*-variables (*n*-wavelengths), PCA aims to find new directions by producing linear combinations of the original combinations. The first component (PC1) corresponds to the direction explaining the maximum variance, while PC2 is the direction, orthogonal to PC1, explaining the maximum variance not explained by PC1, and so on. The score plot (the scores being the coordinates of the samples in the new space, defined by the PCs), allows us to visualize the location of the samples in the space described by the PCs, by making it possible to check similarities and differences among the samples. Standard normal variate (SNV) transform coupled with autoscaling has been performed on the spectral data to remove the multiplicative effects of scattering and to scale the data, respectively [24].

Data analysis was performed by the Chemometric Agil Tool (CAT), an R-based chemometric software developed and implemented by the Chemistry Group of the Italian Chemical Society [30].

## 4. Conclusions

In the present work, the raw diffuse reflectance spectra, coupled with multivariate statistical processing of the data, were proven to be a good strategy for monitoring the color variation in basil semi-finished products during their shelf-life, describing the whole phenomenon. Color is a very important organoleptic feature for food products. It represents a quality control feature of foods that makes a strong first impression upon the consumer. If the color does not produce a positive effect, the food is considered negative, even if its aroma and nutritional values are good.

For these reasons, as concerns the basil semi-finished products that are the object of this study, color represents a fundamental requirement for the acceptability of these products by the manufacturers of the pesto sauce as the final product.

Indeed, DOP Genoese basil is appreciated precisely for its peculiar bright green color, which differentiates it from other varieties of basil, which have a darker and more intense color. Maintaining this color, even during the shelf-life of pesto, is a fundamental quality parameter.

In this work, a simple, rapid, cheap, and non-destructive color analysis (starting from the raw diffuse reflectance spectra of the samples), coupled with an adequate multivariate statistical analysis, proved to be an efficient marker that is usable in the production quality control of these basil semi-finished products.

As regards the modifications to the formulation of the basil semi-finished products, suggested by the company, the greater effect was related to the temperature treatment (blast chilling), followed by the additive effect (ascorbic acid, citric acid, or the mixture of ascorbic acid and citric acid) and the ageing effect (t0, t1, and t2). The blast chilling led to a brighter and more yellow color of the product (higher values of L* and b* CIELab parameters). Through the process of ageing, the samples tended to be browner (higher values of a*), and the citric acid addition in the formulation resulted in a worse color than the use of ascorbic acid.

In conclusion, from the preliminary results obtained in this study, blast chilling, together with the use of ascorbic acid, proved to be the best solution to preserve the color of these products during their shelf-life. Further experimental tests to evaluate other potential formulation changes to these basil semi-finished products are in progress.

## Figures and Tables

**Table 1:** Experimental plan: variables and levels.

		LEVELS			
VAR ID	Variable Name	−1	−0.33	0	1
X_1_	Blast Chilling	No			Yes
X_2_	Citric Acid % *	0		50	100
X_3_	Ageing (month)	t0	t1 (1 m)		t2 (3 m)

* Citric acid (%) with respect to ascorbic acid (%) (i.e., level −1 corresponds to 0% citric acid and 100% ascorbic acid).

**Table 2:** Experimental plan in the standard order.

Exp.	Actual Values			Coded Values		
	Blast Chilling	CitrAc (%)	Time-Point	Blast Chilling	CitrAc (%)	Time-Point
1	Blast Chill	0	t0	1	−1	−1
2	None	0	t0	−1	−1	−1
3	Blast Chill	100	t0	1	1	−1
4	None	100	t0	−1	1	−1
5	Blast Chill	50	t0	1	0	−1
6	None	50	t0	−1	0	−1
7	Blast Chill	0	t1 (1 month)	1	−1	−0.33
8	None	0	t1 (1 month)	−1	−1	−0.33
9	Blast Chill	100	t1 (1 month)	1	1	−0.33
10	None	100	t1 (1 month)	−1	1	−0.33
11	Blast Chill	50	t1 (1 month)	1	0	−0.33
12	None	50	t1 (1 month)	−1	0	−0.33
13	Blast Chill	0	t2 (2 months)	1	−1	1
14	None	0	t2 (2 months)	−1	−1	1
15	Blast Chill	100	t2 (2 months)	1	1	1
16	None	100	t2 (2 months)	−1	1	1
17	Blast Chill	50	t2 (2 months)	1	0	1
18	None	50	t2 (2 months)	−1	0	1

**Table 3:** The basil semi-finished products considered in this study.

Sample Code	Antioxidant Agent	Preliminary Treatment
AscAcid None	L-ascorbic acid (100%)	None
AscAcid_BlastChilling	L-ascorbic acid (100%)	Blast chilling
CitrAcid None	Citric acid (100%)	None
CitrAcid BlastChill	Citric acid (100%)	Blast chilling
AscAcid-CitricAcid None	L-ascorbic acid 50% + citric acid 50%	None
AscAcid-CitricAcid BlastChill	L-ascorbic acid 50% + citric acid 50%	Blast chilling

## Data Availability

The data presented in this study are available in the article.

## Sample Availability

Samples of the compounds are not available from the authors.
